# The first record of the genus *Belenois* (Lepidoptera: Pieridae) from China

**DOI:** 10.3897/BDJ.9.e61332

**Published:** 2021-01-18

**Authors:** Si-Xun Ge, Shao-Ji Hu, Hong-Liang Shi, Feng-Ying Han, Ming-Jun Li, Li-Li Ren

**Affiliations:** 1 College of Forestry, Beijing Forestry University, Beijing, China College of Forestry, Beijing Forestry University Beijing China; 2 Institute of International Rivers and Eco-security, Yunnan University, Kunming, China Institute of International Rivers and Eco-security, Yunnan University Kunming China; 3 Tongliao forest pest control and quarantine station, Tongliao, China Tongliao forest pest control and quarantine station Tongliao China; 4 Kuntouhe foresty centre, Chifeng, China Kuntouhe foresty centre Chifeng China

**Keywords:** *Belenois aurota*, Tibet, new record, high altitude distribution, migratory species

## Abstract

**Background:**

The family Pieridae is a large group of butterflies which plays an important role in evolutionary biology and contains many potential pests (Courtney 1986). Pieridae is a cosmopolitan family while the tropics harbour higher species richness. In a very recent expedition to the Chinese-Indian border area in Tibet Autonomous Region, a migratory species, *Belenois aurota* (Fabricius, 1793), was discovered for the first time, which comprises the first record of the genus *Belenois* in China and the highest altitude record of this species.

**New information:**

The species *B. aurota* (Fabricius, 1793) is the first record of the genus *Belenois* from China. The specimen was collected at an altitude of about 3,000 m in Tibet Autonomous Region. Relevant details are presented for the species.

## Introduction

The family Pieridae, which is a cosmopolitan family, includes over 1,000 species in 83 genera (Ackery et al. 1999, Braby 2005). The adults are of medium size and always sobre colour, typically white or yellow and with black or red patterns. Some species exhibit seasonal phenotypic variation (Braby 2005, Shou et al. 2006).

The genus *Belenois* Hübner, [1819] includes 29 species which are mainly found in the Ethiopian Region and there is only one species (*Belenois java*) that is distributed in the Oriental and Australian Realms. China is a country with mega-biodiversity, with new taxa and new records of butterflies discovered nearly every year in recent times (Hu and Zhang 2009, Hu and Zhang 2010, Hu et al. 2012a, Hu et al. 2012b, Hu et al. 2014, Hu et al. 2018, Zhang and Hu 2018, Zhang et al. 2020a, Zhang et al. 2020b). Even so, a large proportion of Chinese territory is still insufficiently surveyed.

*Belenois* and *Dixeia* are sister groups and belong to the subtribe Aporiina of the tribe Pierini (Wahlberg et al. 2014). The genus *Belenois* was established by Hübner with *Belenois calypso* as its type-species. This genus has 29 known species which are mainly distributed in Africa and south-west Asia and also recorded in Oceania (Shou et al. 2006). However, there is no report of this genus occurring in China.

In this contribution, a well-known migratory species *B. aurota* (Fabricius, 1793) has been collected from Tibet Autonomous Region. The migration of the African colonies of this species almost every year has received lots of attention. The biology of *B. aurota* is also well studied. *B. aurota* larvae feed on plants of the family Capparidaceae, in particular the genus *Capparis*. Under some circumstances, the population erupts at local scale and defoliates the entire bush of *Capparis*; for example, in the Rwenzori National Park in Uganda, it has been a regular pest on *C. decidua* (*Chandra 1985*). The specimens collected in this contribution are the first record of the genus *Belenois* from China.

## Materials and methods

Photographs of the adult were taken with an interchangeable lens digital camera Olympus E-M1 with the lens M. ZUIKO DIGITAL ED 60 mm F2.8 Macro. After removal, the abdomen was soaked in 10% potassium hydroxide solution at room temperature for about 24 hours and was dissected under a Nikon SMZ18 microscope. The genitalia preparation was photographed by a Nikon D7500 digital camera attached to the microscope. Final plates were prepared in Adobe Photoshop CC (Adobe Systems Inc., San Jose, CA, USA). The specimen examined is deposited in the insect collection, Department of Forest Protection, Beijing Forestry University (BFU), Beijing.

## Taxon treatments

### Belenois (Anaphaeis) aurota

(Fabricius, 1793)

1D570FA8-EC16-50B9-8F38-C650FC7A78EF

#### Materials

- **Type status:** Other material. **Occurrence:** recordedBy: Sixun Ge; individualCount: 1; sex: male; lifeStage: adult; disposition: in collection; **Taxon:** scientificName: *Belenois aurota* (Fabricius, 1793); kingdom: Animalia; phylum: Arthropoda; class: Insecta; order: Lepidoptera; family: Pieridae; genus: Belenois; subgenus: Anaphaeis; specificEpithet: aurota; taxonRank: species; verbatimTaxonRank: sp.; scientificNameAuthorship: (Fabricius, 1793); vernacularName: Pioneer White; taxonomicStatus: accepted; **Location:** country: China; stateProvince: Tibet Autonomous Region; county: Zanda County; locality: East of Diya township; verbatimElevation: 2983 m; verbatimCoordinates: 31°78'83"N, 78°87'88"E; **Identification:** identifiedBy: Sixun Ge; dateIdentified: 2019; **Event:** samplingProtocol: sweep net; year: 2019; month: 7; day: 17; habitat: Desolate valley beside the road; **Record Level:** basisOfRecord: PreservedSpecimen

#### Description

**Figure 1.**
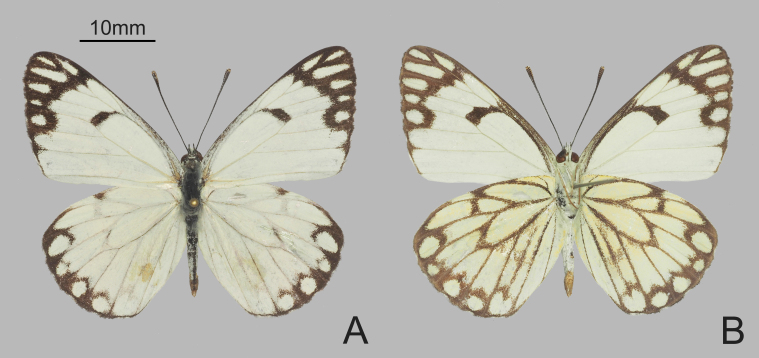
Male *Belenois aurota* (Fabricius, 1793) collected in Diya Township. **A.** upperside; **B.** underside.

**Male** (Fig. 1). Forewing length 44 mm. Body blackish dorsally covered with grey hair and whitish ventrally. Antennae blackish, in typical club shape. Both wings white on the upperside, but with a creamy yellow hue on the underside. Wings with the following markings. Forewing upperside: costa grey, a short black band at the end of the discocell, a curved black subapical band running from costa to vein CuA_1_ and outwardly extending along veins to termen. Forewing underside similar to upperside, but all markings, especially the ones in the apical area, tinged with a golden-brown hue. Hindwing upperside: a black spot at the end of vein Sc+R_1_, termen black fused with a series of subterminal black crescents before vein CuA_2_. Hindwing underside: the previously-mentioned markings, all veins and a short discal band before vein Rs golden-brown.

**Figure 2.**
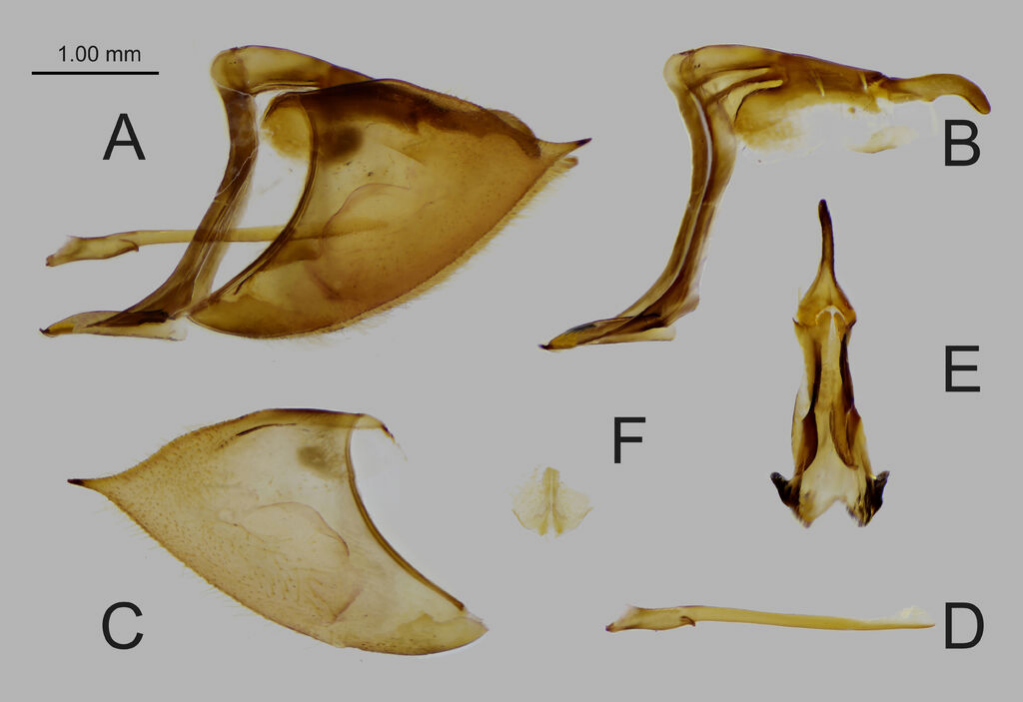
Male genitalia of *B. aurota*. **A.** entire genitalia; **B.** lateral view of ring; **C.** left valve; **D.** lateral view of aedeagus; **E.** dorsal view of tegumen and uncus; **F.** ventral view of juxta.

**Male genitalia** (Fig. 2). Highly sclerotised. Ring straight of moderate width; tegumen narrow in dorsal view; uncus broad at the base, but abruptly narrowed into the same width towards the tip in the dorsal view, while strongly curved ventrally in the lateral view. Saccus slender with a flattened tip. Valve triangulate, slightly elongated distally with an acutely pointed tip, with a long harpe running through the median part. Aedeagus slender and straight, with a pointed tip, Juxta fan-shaped in posterior view.

#### Distribution

South, Southeast and Central Asia; Tropical Africa; China (New record)

## Discussion

*Belenois aurota* is found both in Asia and Africa, this species is migratory, the migration of the species in South Africa has received considerable attention in previous studies (Dickson and Schofield 1958, Taylor 1959), while, except for the populations of the Arabian Peninsula, few records of migration of the Asian populations have been observed; phylogenetic and phylogeographic patterns show that populations of Africa and Asia represent different clusters (Irungbam et al. 2019).

Interestingly, although there has been no official publication of this species in China, we found a specimen labelled “China, Sichuan, Mt. Kintushan, [probably Jinfo Shan in Chongqing] (ohne Datum) [no date], ex coll. A. SCHULTE. Männchen [male].” on a European butterfly website (http://www.euroleps.ch/seiten/s_art.php?art=pier_aurota). We are more inclined to recognise this specimen as a stray butterfly based on its location — far from the known distribution area and a single record for several decades.

**Figure 3.**
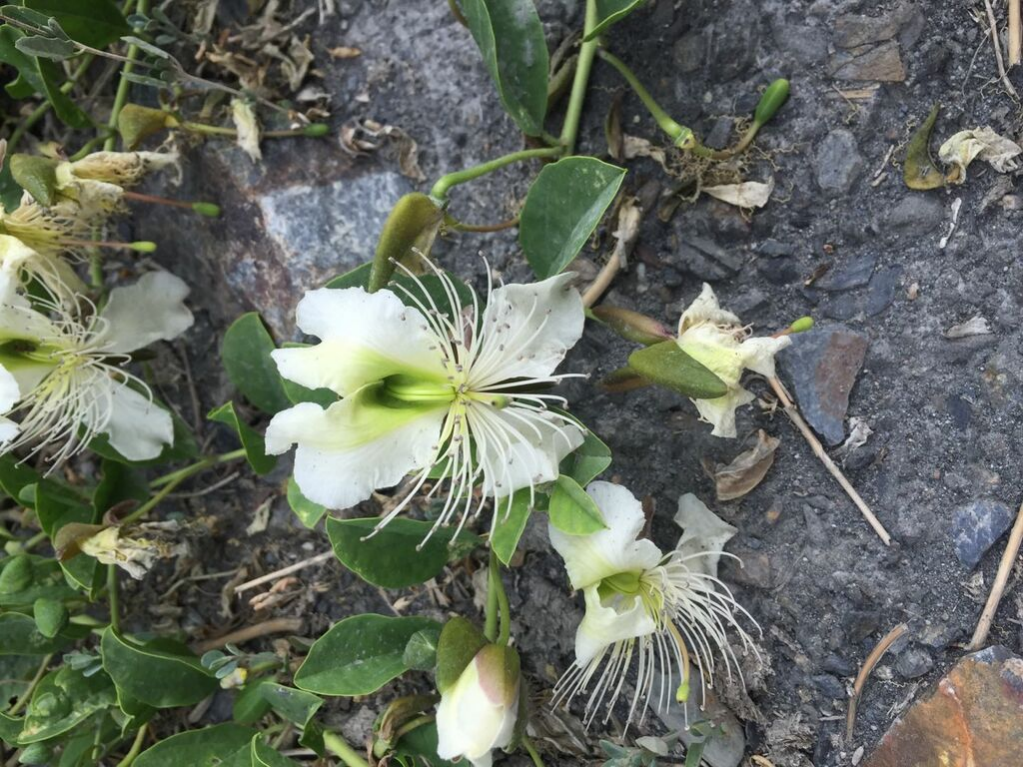
Possible larval food plant, *Capparis himalayensis*, found in Diya Township.

In previous studies, the Himalayan populations of *B. aurota* were mainly distributed in areas below 1,800 m, the highest altitude recorded for the distribution of this species being 2,400 m in Nilgiris, India (Bingham 1907, Cotton et al. 2015). However, in our field investigation, an individual of *B. aurota* was collected at an altitude of about 3,000 m which is the highest altitude record of this species. Colonies of an undetermined *Capparis* species were also found around Diya Township (Fig. 3); we speculate that this species might be *Capparis himalayensis*, which could be a suitable host plant for *B. aurota* larvae. Due to the existence of the host plant, we believe that our *B. aurota* specimen, collected in the Diya Township, was highly likely from a local population rather than a stray butterfly.

## Supplementary Material

XML Treatment for Belenois (Anaphaeis) aurota
